# Bmi1 Is Required for Hepatic Progenitor Cell Expansion and Liver Tumor Development

**DOI:** 10.1371/journal.pone.0046472

**Published:** 2012-09-28

**Authors:** Lingling Fan, Chuanrui Xu, Chunmei Wang, Junyan Tao, Coral Ho, Lijie Jiang, Bing Gui, Shiang Huang, Matthias Evert, Diego F. Calvisi, Xin Chen

**Affiliations:** 1 Department of Bioengineering and Therapeutic Sciences, University of California San Francisco, San Francisco, California, United States of America; 2 Liver Center, University of California San Francisco, San Francisco, California, United States of America; 3 Center for Stem Cell Research and Application, Union Hospital, Tongji Medical College, Huazhong University of Science and Technology, Wuhan, China; 4 School of Pharmacy, Tongji Medical College, Huazhong University of Science and Technology, Wuhan, China; 5 Institute of Pathology, University of Greifswald, Greifswald, Germany; Institute of Hepatology London, United Kingdom

## Abstract

Bmi1 is a polycomb group transcriptional repressor and it has been implicated in regulating self-renewal and proliferation of many types of stem or progenitor cells. In addition, Bmi1 has been shown to function as an oncogene in multiple tumor types. In this study, we investigated the functional significance of Bmi1 in regulating hepatic oval cells, the major type of bipotential progenitor cells in adult liver, as well as the role of Bmi1 during hepatocarcinogenesis using *Bmi1* knockout mice. We found that loss of Bmi1 significantly restricted chemically induced oval cell expansion in the mouse liver. Concomitant deletion of *Ink4a/Arf* in *Bmi1* deficient mice completely rescued the oval cell expansion phenotype. Furthermore, ablation of *Bmi1* delayed hepatocarcinogenesis induced by AKT and Ras co-expression. This antineoplastic effect was accompanied by the loss of hepatic oval cell marker expression in the liver tumor samples. In summary, our data demonstrated that Bmi1 is required for hepatic oval cell expansion via deregulating the *Ink4a/Arf* locus in mice. Our study also provides the evidence, for the first time, that Bmi1 expression is required for liver cancer development *in vivo*, thus representing a promising target for innovative treatments against human liver cancer.

## Introduction

Liver is a unique organ, being silent in normal circumstances but displaying regenerative properties following damage and/or parenchymal loss. Liver regeneration involves two different cellular compartments, depending on the nature of the injury. In response to the acute mass loss injury, such as partial hepatectomy, liver regeneration is due to the proliferation of hepatocytes from the remaining lobes [1]. Under chronic injury conditions that impair hepatocyte proliferation, a subpopulation of unique cells, which has been termed as “oval cells”, emerges and expands. Oval cells are considered to be hepatic stem or progenitor cells because of their bi-potential capability of differentiating into both hepatocytes and cholangiocytes [2], [3]. Several models of oval cell reaction in rodents have been developed by exposing the animals to certain carcinogens, such as 3,5-diethoxycarbonyl-1,4-dihydro-collidine (DDC) [4], carbon tetrachloride (CCl4) [5], and 2-acetylaminofluorene (2-AAF) [6], [7], among others. In these models, oval cells arise from the portal area of the lobule and infiltrate the surrounding parenchyma. The molecular features of oval cells are still controversial, mostly due to the lack of definitive markers to identify these cells. For instance, A6, a monoclonal antibody previously described by Factor VM [8], and Cytokeratins (CKs), such as CK7 and CK19, are widely used as oval cell markers in rodents [9]. Recently, the epithelial cell adhesion molecule (EpCAM) was also found in the oval cell niche [10]. Unfortunately, all of these biomarkers can also stain the bile duct epithelium, thus limiting their usefulness as primary antibodies for oval cell isolation. Recently, a new panel of monoclonal antibodies directed against OC2s by immunization of rats with enzymatically dispersed non-parenchymal cells from the DDC-treated mouse livers in searching for oval cell specific antigens have been developed. However, whether these antibodies truly recognize oval cells or whether they recognize different oval cell sub-populations remains to be fully explored [11].

Hepatocellular carcinoma (HCC) and cholangiocarcinoma (CC) are the two most common types of primary liver tumors, which originate from hepatocytes and cholangiocytes, respectively. However, some primary liver cancer showed intermediate or combined (HCC/CC) phenotypes. Those tumors were thought to be originated from transformed progenitor (oval) cells [12], [13], [14]. It has been hypothesized that maturation arrest might occur when a bi-potential progenitor cell is on its way to differentiation, which can give rise to tumors with a range of phenotypes with heterogeneous HCC and CC features [13]. In a previous study, we developed a mouse model in which co-activation of AKT and N-Ras oncogenes results in rapid development of HCC and CC in the mouse liver [15].

Bmi1 was initially identified as a c-Myc cooperating oncogene in murine B-cell lymphomas [16], and subsequently determined to be a member of the Polycomb group of transcriptional repressors [17], [18], which participate in regulating cell cycle and senescence. Using knockout mice, it was found that Bmi1 deletion results in neurological abnormalities and severe hematopoietic defects in mice [19]. Subsequent studies revealed that Bmi1 is essential for self-renewal of both normal and leukemic haematopoietic stem cells [20], [21], as well as neural stem cells [22]. Recently, the essential role of Bmi1 as an oncogene has been revealed in multiple tumor types, including breast cancer [23], melanoma [24], prostate cancer [25], non-small cell lung carcinomas [26], [27] and HCC [28], [29]. The *Ink4a/Arf* locus was identified as a critical downstream target of Bmi1. In mice, *Ink4a/Arf* encodes p16^Ink4a^ and p19^Arf^ genes, and both are important tumor suppressors. Of note, p16^Ink4a^ regulates cell cycle progression via modulating Cdk4/cyclin D complexes, whereas p19^Arf^ regulates cell apoptosis via the MDM2/p53 pathway. Recent studies have demonstrated that Bmi1, together with other polycomb proteins, binds throughout the *Ink4a/Arf* locus, and represses p16^Ink4a^ and p19^Arf^ expression [30]. Furthermore, it has been shown that ablation of *Ink4a/Arf* dramatically reduced the lymphoid and neurological defects in *Bmi1* deficient mice [31]. However, *Bmi1* and *Ink4a/Arf* double knockout mice remain small and unfertile, similar to that observed in *Bmi1* knockout mice [32], indicating the existence of additional *Ink4a/Arf* independent regulatory pathways. Consistent with this hypothesis, a recent study suggested that Bmi1 also plays a role in the regulation of mitochondrial function and the DNA damage response pathway [33]. In particular, it has been shown that treatment with the antioxidant N-acetylcysteine (NAC) reduced the elevated reactive oxygen species (ROS) characteristic of *Bmi1* deficient mice. Consistently, NAC rescued the defects in thymocyte maturation in *Bmi1* null mice.

Although Bmi1 is known to play critical roles in regulating multiple types of stem or progenitor cells, its functional significance in regulating hepatic oval cells and hepatocarcinogenesis remains poorly understood. In the present study, using *Bmi1* null mice, we demonstrated that Bmi1 is required for DDC-induced oval cell expansion *in vivo*. To investigate the molecular mechanism underlying this phenotype, we evaluated the oval cell expansion in *Bmi1* and *Ink4a/Arf* double knockout mice as well as *Bmi1* null mice treated with NAC. Our study clearly demonstrates that loss of *Ink4a/Arf* rescues the oval cell expansion defects in *Bmi1* null mice, supporting the hypothesis that Bmi1 regulates hepatic oval cells via modulation of the *Ink4a/Arf* locus. Furthermore, we co-expressed activated forms of AKT and Ras in *Bmi1* null mice to evaluate the role of Bmi1 in hepatocarcinogenesis. The results indicate that ablation of *Bmi1* dramatically delays liver tumor development driven by AKT and Ras co-expression. Delayed hepatocarcinogenesis was accompanied by the loss of hepatic oval cell marker expression in the AKT/Ras liver tumor samples. Altogether, our study provides novel insights into the role of Bmi1 in regulating hepatic progenitor cell proliferation and hepatocarcinogenesis.

## Results

### Bmi1 is expressed in hepatic oval cells and is required from oval cell expansion

**Figure 1:**
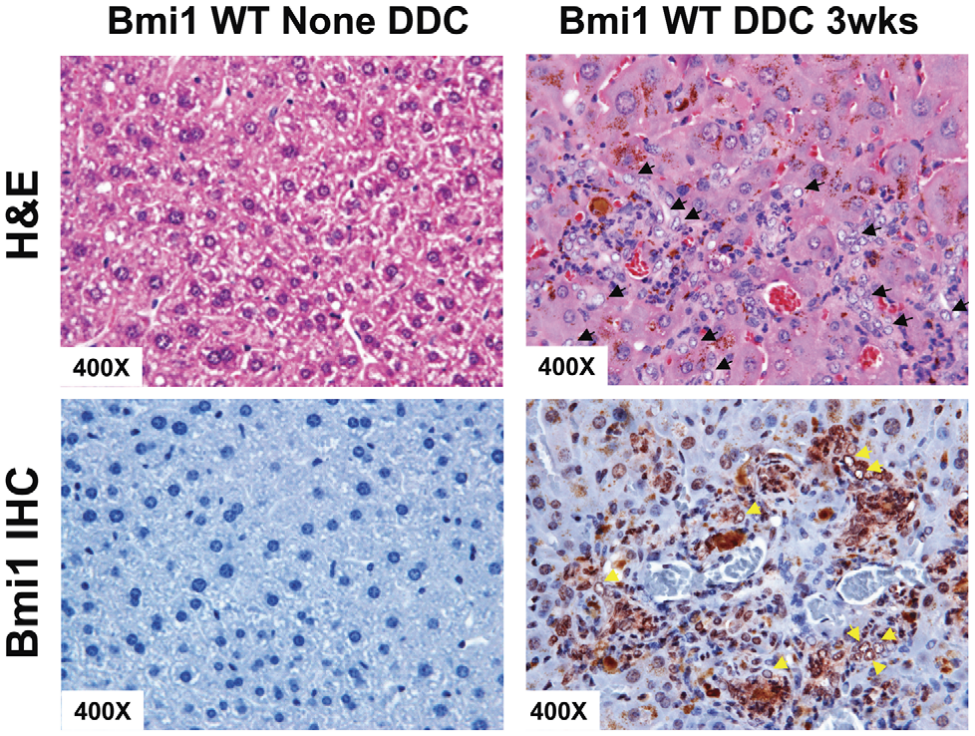
Hepatic oval cell expresses Bmi1. H&E staining (upper row) and immunostaining of Bmi1 (lower row) in the wildtype (left) and DDC treated (right) mouse liver tissues. Arrows indicate expanding oval cells. At least 3 animals in each group were assayed.

Despite the fact that Bmi1 is considered to be an important stem cell marker, it remains unknown whether Bmi1 is expressed in hepatic oval cells. We therefore investigated the expression of Bmi1 in hepatic oval cells. To establish a stable oval cell expansion model for this study, adult wild-type mice were randomized to normal diet or DDC diet for 3 weeks. Consistent with the previous reports, typical histological changes were detected in all DDC treated mouse livers. H&E staining revealed a population of small cells with a large nucleus to cytoplasm ratio in the periportal area of the liver lobule, in the DDC treated mouse livers. Many of these small cells had an atypical duct-like morphology, which is a well-known oval cell phenotype [4], [37] (Figure 1). Immunohistochemical staining showed the nuclear Bmi1 staining in these oval cells (Figure 1 and Figure S1). In contrast, Bmi1 expression was undetectable in normal liver tissues (Figure 1). Consistent with these data, Bmi1 mRNA level was higher in DDC treated liver tissues compared with that in untreated liver tissues (Figure S2).

**Figure 2:**
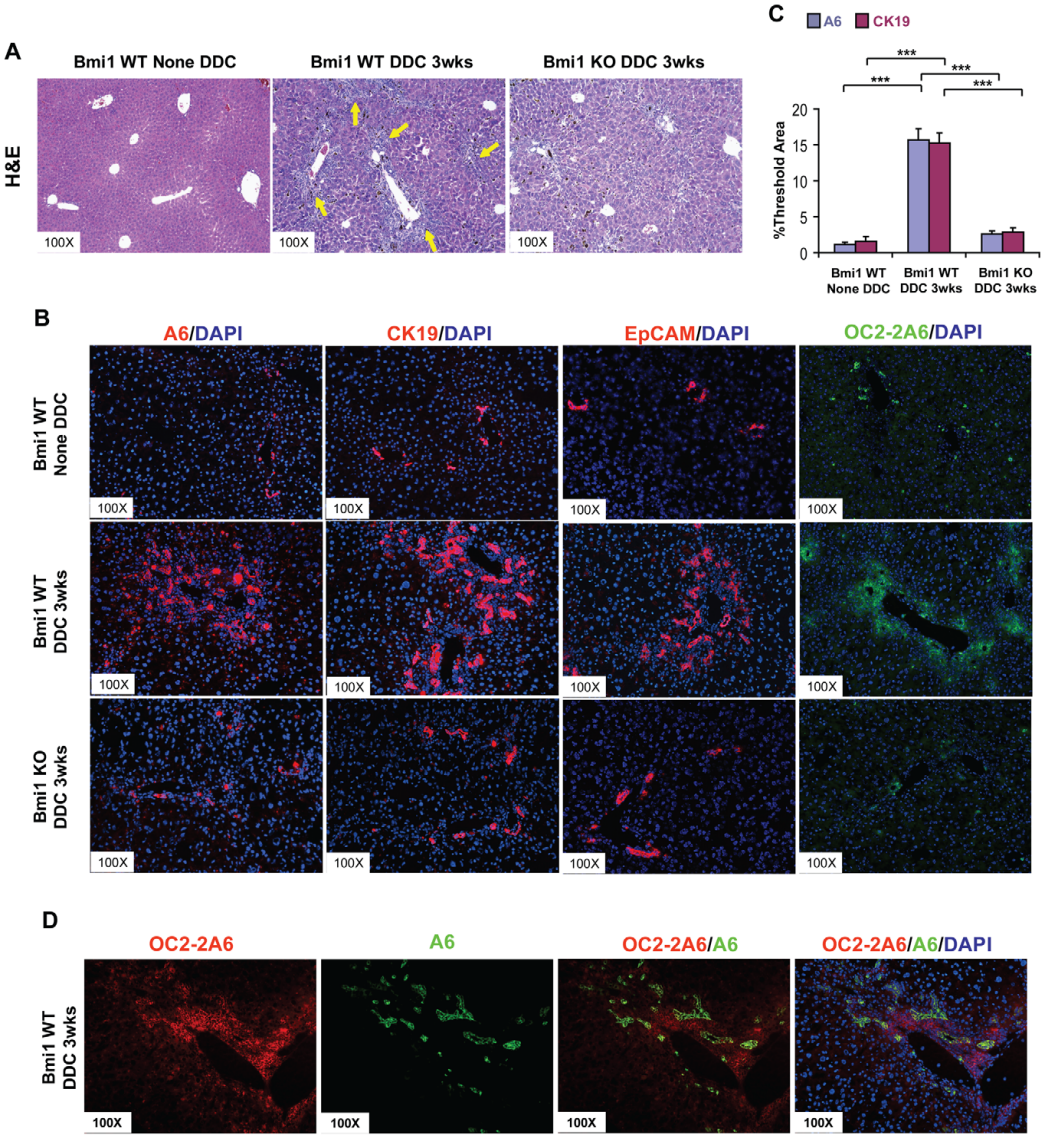
Loss of Bmi1 inhibits DDC induced hepatic oval cell expansion in mice. H&E staining (A) and immunostaining of A6, CK19, EpCAM and OC2-2A6 (B) on sections of Bmi1 wild-type (WT) liver without DDC treatment; Bmi1 WT liver with 3wk-DDC treatment; and Bmi1 knockout (KO) liver with 3wk-DDC treatment. Representative images of three independent experiments are shown. Expanded views of A6 and CK19 staining are shown on the lower right corner; (C) Quantification of A6 and CK19 immunostaining using ImageJ software. ***P<0.001 by Student's *t* test. (D) Dual staining of OC2-2A6 (periductal) and A6 (ductal) showing different oval cell populations in DDC treated mouse liver. Yellow arrows in (A) represents expanded oval cells. At least 3 animals in each group were assayed.

Next, we subjected *Bmi1^−/−^* mice (n = 5) and their littermates with *Bmi1^+/+^* or *Bmi1^+/−^* genotypes (n = 9) to the DDC treatment. Oval cell expansion could be clearly visualized in DDC treated *Bmi1^+/+^* or *Bmi1^+/−^* mice (Figure 2A and data not shown). By immunofluorescence staining, we detected positive staining for the ductal oval cell markers A6, CK19 and EpCAM in both untreated and DDC treated mouse livers (Figure 2B). However, in the untreated liver sections, cells positive for A6, CK19 and EpCAM markers were limited to bile duct cells in the periportal region. In DDC treated livers, A6-, CK19-, and EpCAM-stained cells were characterized by atypical ductal proliferation, and stretched from the periportal area to the central area (Figure 2B). In addition, the extensive staining of periductal marker OC2-2A6 without overlapping with ductal marker A6 (Figure 2D) further confirmed the oval cell expansive pattern. In striking contrast, we found that the oval cell expansion was significantly reduced in DDC treated *Bmi1^−/−^* mouse livers (Figure 2A). Few atypical duct-like cells were detected in *Bmi1^−/−^* mice. Using A6 and CK19 staining, we found that areas positive for of A6 and CK19 staining were significantly decreased in the DDC treated *Bmi1^−/−^* livers, when compared with *Bmi1^+/+^* controls (Figure 2B and 2C). Similar results were obtained with EpCAM (Figure 2B). Interestingly, we observed the loss of the periductal cell marker OC2-2A6 in the DDC treated *Bmi1^−/−^* livers (Figure 2B), suggesting the defective expansion of these periductal cells in *Bmi1* deleted mice.

Altogether, our results provide solid evidence that Bmi1 is expressed in hepatic oval cell population, and ablation of Bmi1 significantly inhibits the oval cell expansion induced by DDC treatment. The data indicate that Bmi1 is essential for hepatic oval cell expansion in the DDC mouse model.

### Antioxidant treatment does not rescue the oval cell expansion in *Bmi1^−/−^* Mice

**Figure 3:**
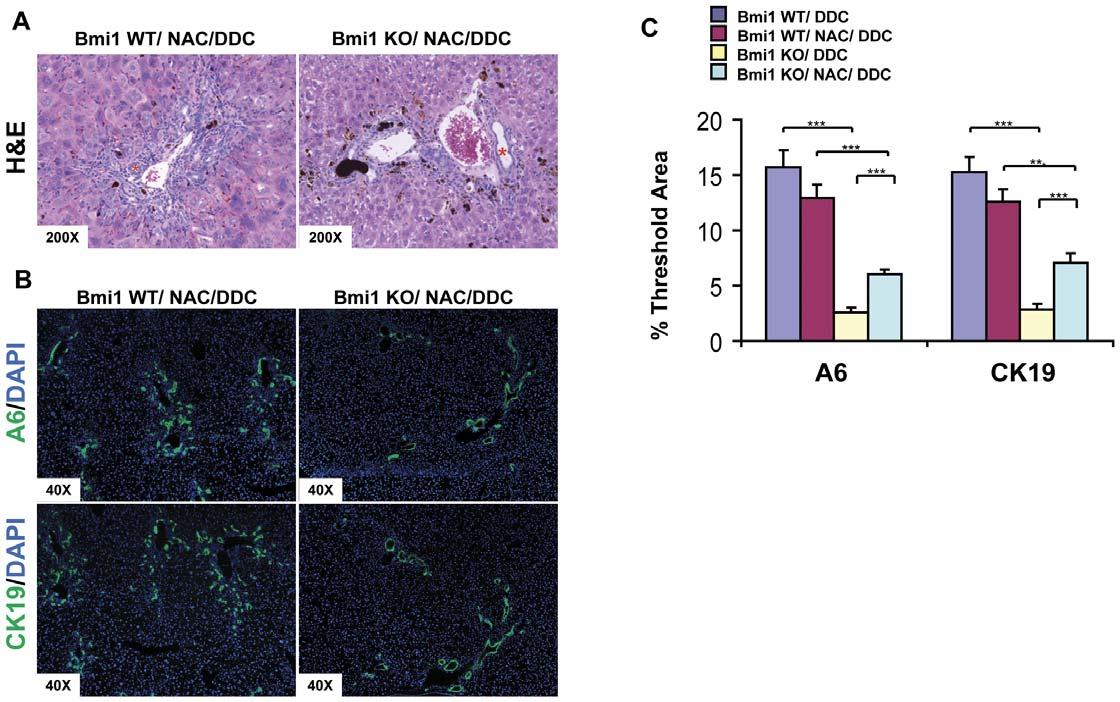
Antioxidant treatment enlarges bile ducts but does not rescue the oval cell expansion defects in *Bmi1^−/−^* Mice. H&E staining (A) and A6 and CK19 immunostaining (B) on *Bmi1* WT and *Bmi1* KO DDC livers subjected to the treatment with the NAC antioxidant. Representative images of three independent experiments are shown. Asterisk * in (A) represents an enlarged bile duct. (C) Quantification of A6 and CK19 staining on *Bmi1* WT or KO DDC livers without or with NAC treatment using ImageJ software. At least 3 animals in each group were assayed. **P<0.01, ***P<0.001 by Student's *t* test.

**Figure 4:**
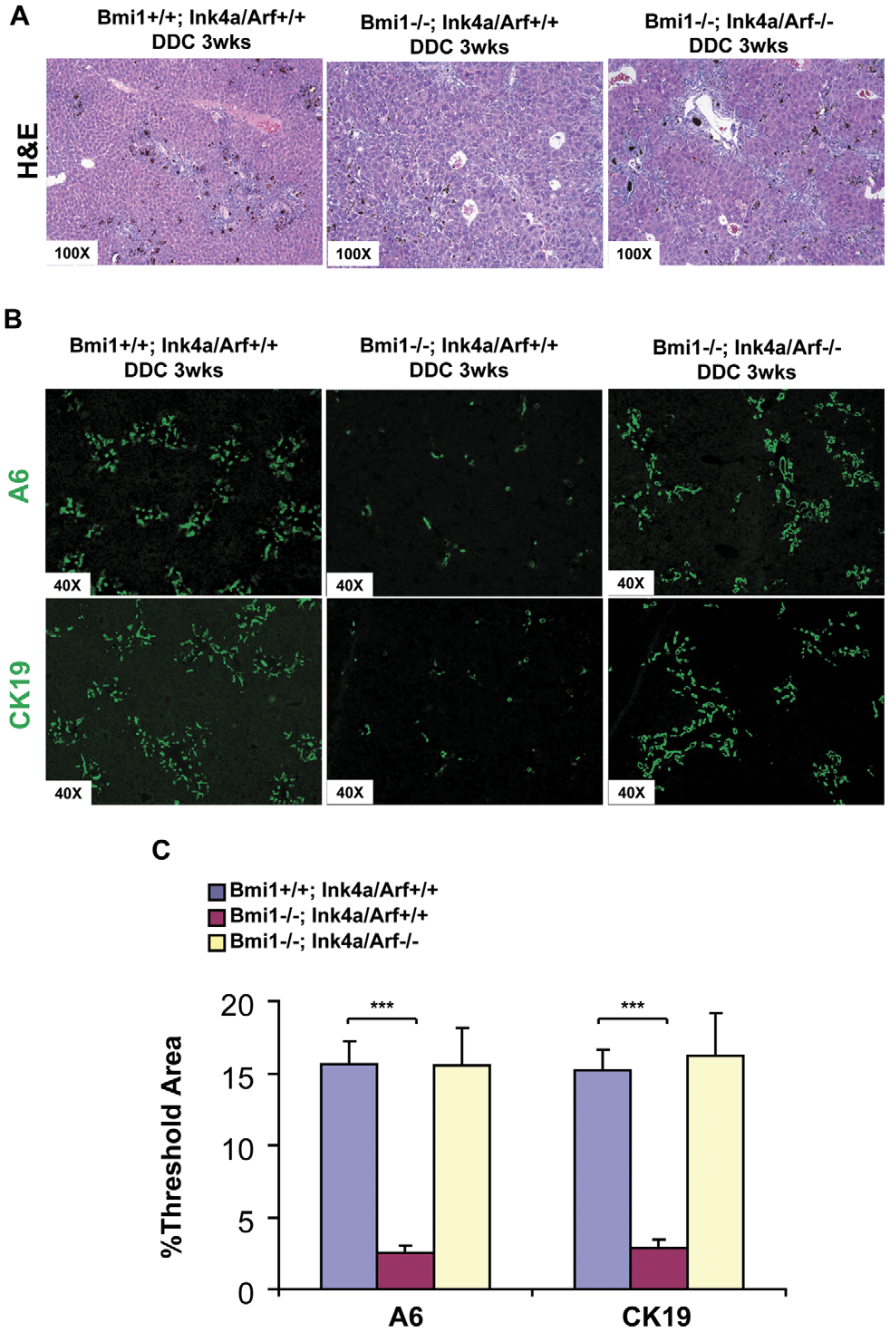
Deletion of *Ink4a/Arf* rescues oval cell expansion defects in *Bmi1^−/−^* mice. H&E staining (A) and A6 and CK19 immunostaining (B) on *Bmi1^+/+^; Ink4a/Arf^+/+^*, *Bmi1^−/−^; Ink4a/Arf^+/+^* and *Bmi1^−/−^; Ink4a/Arf^−/−^* DDC treated mouse livers. Representative images of three independent experiments are shown. (C) Quantification of A6 and CK19 staining using ImageJ software. At least 3 animals in each group were assayed. ***P<0.001 by Student's *t* test.

Next, we investigated the molecular mechanisms that underlie the Bmi1 mediated hepatic oval cell expansion. It has been reported that increased ROS levels contribute to the multiple *in vivo* defects observed in *Bmi1^−/−^* mice [33]. To examine whether Bmi1 regulates oval cell expansion via the ROS pathway, we performed pharmacological treatment with the antioxidant NAC as previously described [33]. We found that there was an increased fluorescent signal of A6 and CK19 in the *Bmi1^−/−^* DDC livers supplemented with NAC when compared with untreated *Bmi1^−/−^* DDC livers (Figure 3C). However, the typical oval cell expansion phenotypic appearance that could be readily observed in *Bmi1^+/+^* DDC livers (n = 8) was not apparent in the NAC treated *Bmi1^−/−^* DDC livers (n = 5) (Figure 3A and 4B). Intriguingly, morphological evaluation of the livers revealed that an enlargement of bile ducts occurred in response to NAC treatment (Figure 3A), which was presumably the cause of the increased overall fluorescent signal for A6 and CK19 antibodies in these mice. Consistent with this result, lipid peroxidation which is commonly used as the indicator of oxidative stress in tissues, assessed by MDA content, was not elevated in liver tissues from DDC treated *Bmi1^−/−^* mice when comparing with liver tissues from DDC treated wildtype mice (Figure S3). Altogether, our data suggest that the role of Bmi1 in oval cell expansion is most likely unrelated to the role of Bmi1 in regulating mitochondrial function and ROS levels.

### *Ink4a/Arf* deletion completely rescues the oval cell expansion in *Bmi1^−/−^* mice

The *Ink4a/Arf* locus is a well-characterized downstream target of Bmi1. Indeed, we found that expression levels of p16Ink4A and p19Arf were higher in liver tissues from DDC treated *Bmi1^−/−^* mice compared with liver tissues from DDC treated wildtype mice (Figure S4). We therefore hypothesized that Bmi1 regulates hepatic oval cell expansion in an *Ink4a/Arf*-dependent manner. To test this hypothesis generated *Bmi1* and *Ink4a/Arf* double knockout mice (*Bmi1^−/−^; Ink4a/Arf^−/−^*) to explore whether the deletion of *Ink4a/Arf* locus could rescue the defective oval cell expansion phenotype observed in *Bmi1^−/−^* mice.

As a first step, we determined whether *Ink4a/Arf* locus affects oval cell expansion. For this purpose, we compared the oval cell expansion in *Ink4a/Arf^+/+^* DDC livers and *Ink4a/Arf^−/−^* DDC livers. Using H&E morphological analysis as well as immunofluorscence staining for A6 and CK19, we found similar oval cell expansion phenotypes in *Ink4a/Arf^+/+^* and *Ink4a/Arf^−/−^* mice (data not shown), indicating that loss of *Ink4a/Arf* locus has no effect *per se* on oval cell proliferation. Subsequently, we generated *Bmi1^−/−^;Ink4a/Arf^−/−^* mice and treated the mice with DDC diet (n = 4). Histological analysis and A6 and CK19 staining showed that, when compared to the *Bmi1^−/−^; Ink4a/Arf^+/+^* DDC livers (n = 3), the typical oval cell expansion pattern was restored in *Bmi1^−/−^; Ink4a/Arf^−/−^* DDC livers, with a similar labeling pattern observed in *Bmi1^+/+^;Ink4a/Arf^+/+^* (n = 4) DDC livers (Figure 4A and 4B). Quantification analysis demonstrated that the percentage of A6 and CK19 positive areas on *Bmi1^−/−^;Ink4a/Arf ^−/−^* DDC livers was restored to the same level of *Bmi1^+/+^;Ink4a/Arf^+/+^* DDC liver sections (Figure 4C).

In summary, the present results indicate that the ablation of *Ink4a/Arf* locus in *Bmi1* deficient mice can completely restore the oval cell expansion. Therefore, our *in vivo* study demonstrates that Bmi1 plays its essential role in hepatic oval cell proliferation in an *Ink4a/Arf-*dependent manner.

### Ablation of Bmi1 inhibited AKT/Ras induced hepatocarcinogenesis

**Figure 5:**
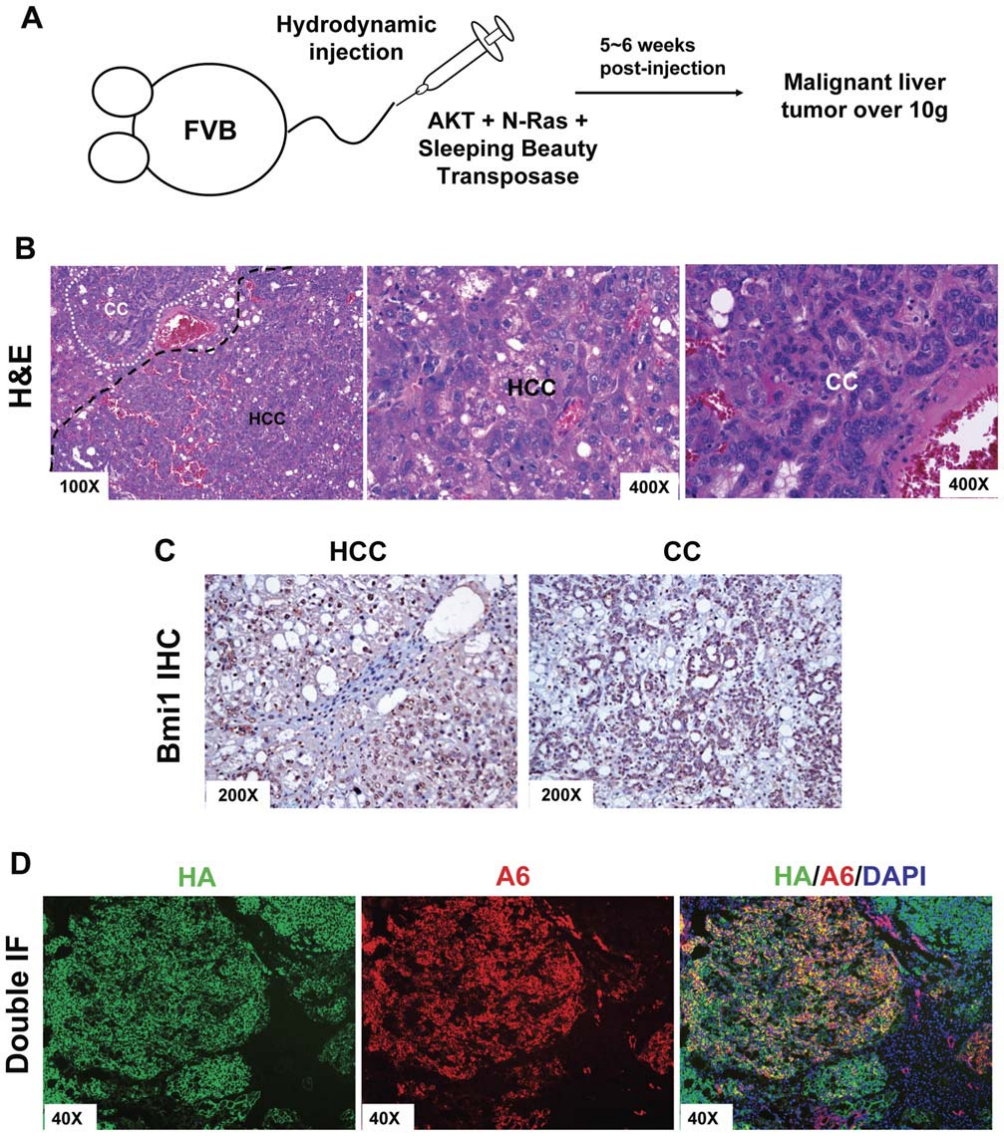
Co-expression of myristylated AKT1 (myr-AKT) and mutated N-Ras (N-RasV12) induces mouse liver tumor development. (A) Depiction of the mouse model generation using hydrodynamic injection. (B) H&E staining of AKT/Ras injected mouse liver (6 weeks post injection). Representative images of three independent experiments are shown. (C) Bmi1 expression in Wildtype liver and AKT/Ras induced HCC and CC tumor samples. (D) Double immunofluorescence staining of HA-tag and A6 on AKT/Ras injected mouse liver. AKT/Ras injected mouse liver (6 weeks post injection). At least 3 animals in each group were assayed. Representative images of three independent experiments are shown.

In a recent study, we developed a novel liver tumor model by co-expressing myr-AKT (with C-terminal HA tag) and N-RasV12 genes via hydrodynamic injection [38], which will be referred to as AKT/Ras mice in this paper. In this mice model, co-activation of AKT and Ras rapidly induced liver tumors in five to six weeks after injection (Figure 5A). Histological evaluation of the liver showed that both HCC, representing ∼70% of the liver lesions and CC, ∼30% of the liver lesions, were present in AKT/Ras livers (Figure 5B), in accordance with our previous report [15]. Because AKT/Ras induced both HCC and CC, we investigated whether the oval cell marker A6 was expressed in these tumor cells. We found that double staining of HA-tag, which labeled ectopically injected AKT, and A6 consistently co-localize in HCC and CC lesions (Figure 5D). These data, therefore, suggest that AKT/Ras hepatocarcinogenesis might depend on hepatic progenitor cells.

Loss of *Bmi1* has been previously shown to inhibit the tumor development in multiple mouse models, such as K-Ras induced lung cancer [38] or hedgehog pathway-driven medulloblastoma [39]. In addition, the present study supports the critical role of Bmi1 in regulating A6 positive liver progenitor cell expansion. Thus, we sought to determine whether Bmi1 expression is required for AKT/Ras induced liver tumor development. As the first step, we evaluated Bmi1 expression in AKT/Ras tumor cells, and we found that expression of Bmi1 in both HCC and CC lesions (Figure 5C). Hydrodynamic injection of AKT/Ras into *Bmi1^−/−^* mice (n = 6) as well as *Bmi1^+/+^* control littermates (n = 5) was performed (Table 1). In all the control mice, massive abdomen enlargement was evident within 4 weeks after injection and mice became moribund, requiring to be euthanized by 6 to 7 weeks (Table 1). In contrast, all *Bmi1^−/−^* mice appeared to be normal with no palpable abdominal mass 6 to 8 weeks post-injection (Table 1). Unfortunately, we were unable to maintain the AKT/Ras injected *Bmi1^−/−^* mice beyond 8 weeks post injection since these mice started to die due to infections consequent to their severe immunodeficiency.

**Table 1:** Ablation of *Bmi1* inhibits AKT/Ras induced hepatocarcinogenesis.

Code	Genotype	Gender	W.P.I (*)	liver weight	body weight	Ratio (#)
WT AKTRas 1	Bmi1+/+	Male	5	13.3	38.9	0.34
WT AKTRas 2	Bmi1+/+	Male	5	13.8	37.2	0.37
WT AKTRas 3	Bmi1+/+	Female	6	8.8	31.3	0.28
WT AKTRas 4	Bmi1+/+	Female	6	8.6	28.3	0.30
WT AKTRas 5	Bmi1+/+	Female	7.5	13.3	35.1	0.38
KO AKTRas 1	Bmi1−/−	Male	8	1.2	19.3	0.06
KO AKTRas 2	Bmi1−/−	Male	8	1.1	15.4	0.07
KO AKTRas 3	Bmi1−/−	Male	8	1.1	16.2	0.07
KO AKTRas 4	Bmi1−/−	Female	6	0.5	8.7	0.06
KO AKTRas 5	Bmi1−/−	Female	8	1	17.7	0.06
KO AKTRas 6	Bmi1−/−	Female	8	0.9	11.5	0.08

* refers to weeks post injection; # refers to the ratio of liver weight to body weight.

**Figure 6:**
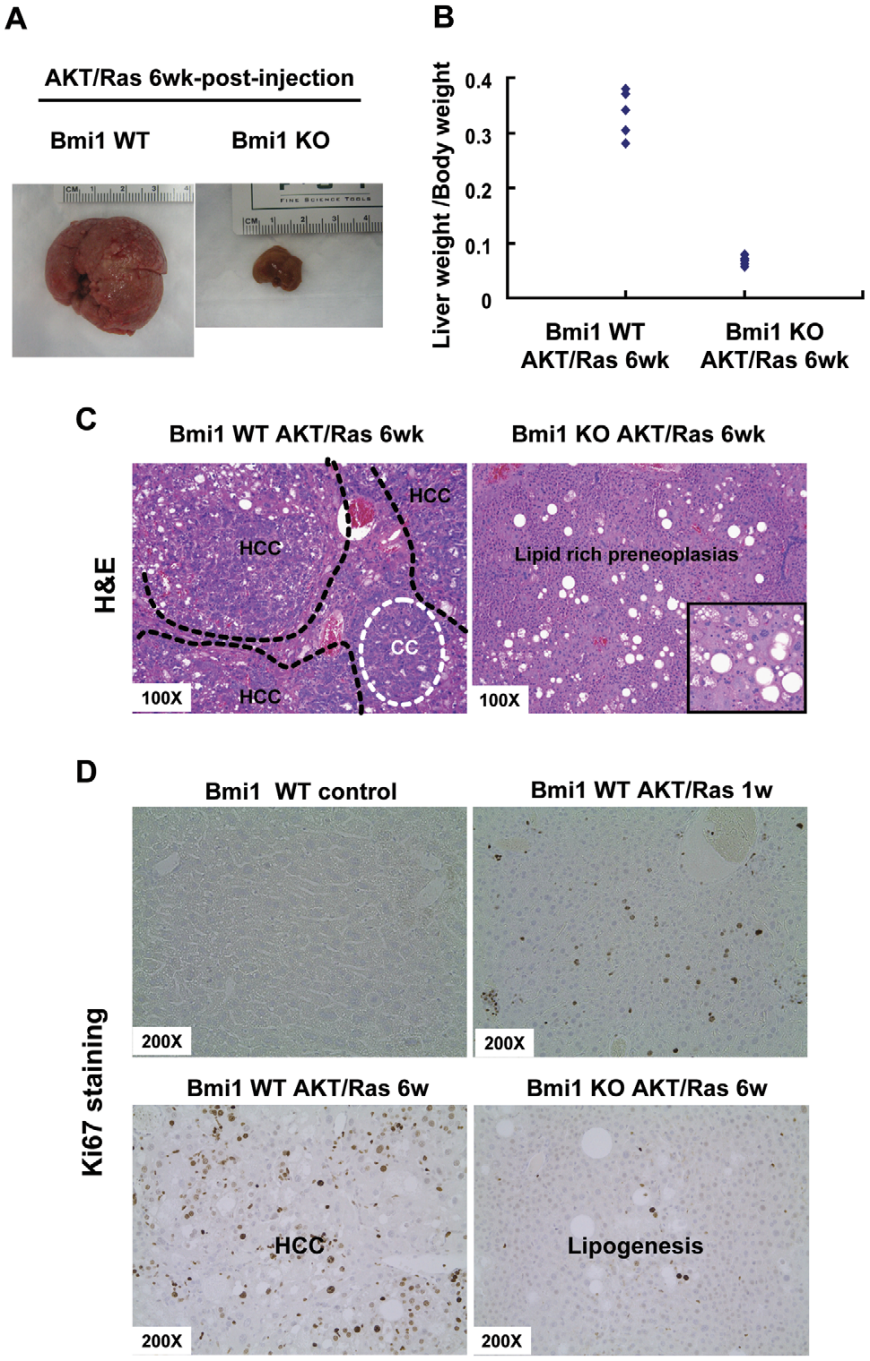
Absence of *Bmi1* decelerates AKT/Ras induced liver tumor development. Gross images (A), Ratio of liver to body weight (B), H&E staining (C) and Ki67 staining (D) of *Bmi1* WT AKT/Ras liver and *Bmi1* KO AKT/Ras liver (6 weeks post injection). At least 3 animals in each group were assayed. Representative images of three independent experiments are shown.

Upon dissection, tumor nodules were present throughout the liver of AKT/Ras injected wild-type mice (Figure 6A). The average liver weight was ∼11.6 g, and the liver to body ratio of approximately 0.34 in AKT/Ras wild-type mice (Table 1 and Figure 6B). In contrast, no visible nodular lesions could be identified in the livers from AKT/Ras injected *Bmi1^−/−^* mice, although the liver appeared to be paler and spottier than normal liver (Figure 6A). Liver weight average was approximately 1 g, and liver to body ratio ∼0.06 in AKT/Ras injected *Bmi1^−/−^* mice (Table 1 and Figure 6B).

Histological analysis revealed that neoplastic lesions occupied the majority of the liver parenchyma in AKT/Ras injected wild-type mice. Tumors showed both HCC and CC phenotypes, in accordance with our previous report (Figure 6C) [15]. In contrast, only lipid-rich hepatocytes could be observed, and no neoplastic lesions, such as HCC or CC were present in AKT/Ras injected *Bmi1^−/−^* mice (Figure 6C). The lipogenic hepatocytes likely accounted for the pale and spotty appearance of the liver from AKT/Ras injected *Bmi1^−/−^* mice.

To investigate the molecular mechanism underlying this phenotype, we assayed the cell proliferation rate in normal liver, AKT/Ras injected wild-type or *Bmi1^−/−^* mice. In normal liver, few Ki67 positive cells could be observed (Figure 6D). Ki67 positive cells were detected throughout the liver tumor tissues from AKT/Ras injected wild-type mice at 6 weeks post-injection (Figure 6D). In contrast, most lipogenic hepatocytes in AKT/Ras injected *Bmi1^−/−^* mice at 8 weeks post injection showed no or few Ki67 staining (Figure 6D). Since AKT/Ras injected *Bmi1^−/−^* mice did not develop liver tumors, we also assayed Ki67 staining pattern in the preneoplastic liver tissues in the wild-type mice injected with AKT/Ras at 1 week post-injection. At this stage, only lipogenic hepatocytes could be observed in the liver with no tumor lesions, similar to the phenotype observed in AKT/Ras injected *Bmi1^−/−^* mice at 8 weeks post injection. A significant higher numbers of Ki67 positive cells could be found in these preneoplastic liver tissues comparing with that in AKT/Ras injected *Bmi1^−/−^* mice at 8 weeks post injection (Figure 6D). Thus, Bmi1 expression is required for tumor cell proliferation during AKT/Ras induced hepatocarcinogenesis.

**Figure 7:**
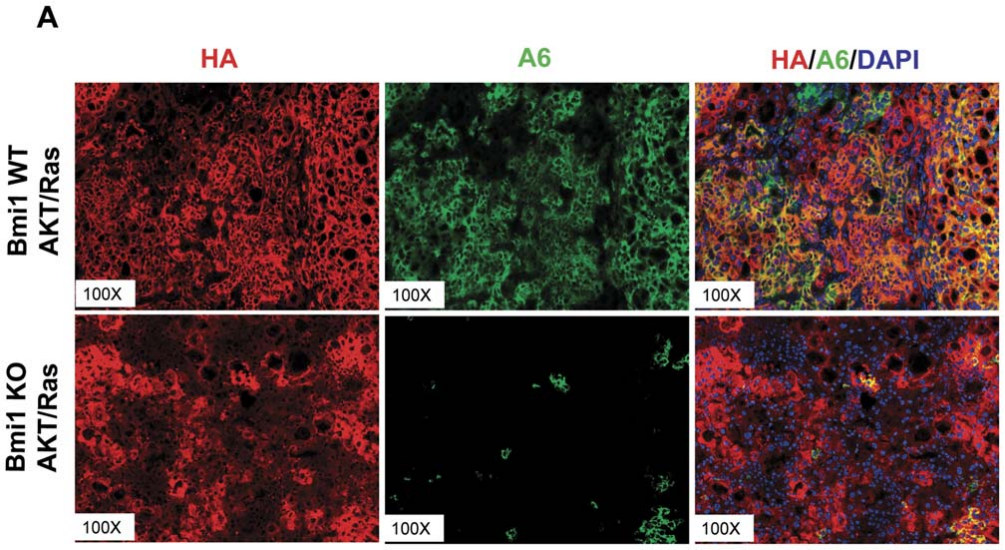
Absence of Bmi1 restricts A6 expression in AKT/Ras liver tumors. Double immunofluorescence staining of HA-tag and A6 on *Bmi1* WT AKT/Ras and *Bmi1* KO AKT/Ras livers. At least 3 animals in each group were assayed.

Double immunofluorscence staining of HA-tag and A6 revealed extensive (greater than 95%) co-localized expression of ectopically injected AKT with A6 in liver tumors from AKT/Ras injected wild-type mice (Figure 7). In contrast, while extensive and strong HA-tag and AKT immunoreactivity could be observed in AKT/Ras injected *Bmi1^−/−^* mouse livers, only very limited A6 expression (less than 5%) was detected (Figure 7).

Altogether, the present data indicate that Bmi1 is required for AKT/Ras driven hepatocarcinogenesis, most likely by regulating hepatic progenitor cell proliferation.

## Discussion

Bmi1, a member of the Polycomb group protein family, is required for maintaining self-renewal of stem or progenitor cell in multiple organs, particularly in the neural and hematopoietic system. In the current study, we showed Bmi1 is expressed in hepatic oval cells and the oval cell expansion is significantly inhibited when *Bmi1* is deleted, supporting the critical role of Bmi1 in regulating this type of liver progenitor cells. In order to investigate the mechanisms whereby Bmi1 influences oval cell expansion and proliferation, we designed two rescue experiments based on the functional roles that have been ascribed to Bmi1. The first mechanism is the regulation of mitochondrial function played by Bmi1 via modulation of ROS levels [33], [40]. To investigate this issue, we treated the mice with the NAC antioxidant, which has been demonstrated to rescue the *Bmi1^−/−^* thymocyte development and other defects. The results showed that the typical hepatic oval cell expansion phenotype was absent in *Bmi1^−/−^* mice treated with DDC and NAC. However, a marked bile duct enlargement phenotype occurred in these mice. This observation indicates that ROS level does not affect hepatic oval cell expansion, but may influence the biliary tract development. The mechanism leading to the bile duct enlargement in response to NAC remains unclear and requires further investigation. As concerns the second mechanism, Bmi1 has been previously shown to modulate stem cell proliferation via repressing the *Ink4a/Arf* locus. For instance, multiple studies have demonstrated that loss of *Ink4a/Arf* locus rescued the hematopoietic stem cell defects [31], [41], [42] and neurological stem cell abnormalities [32], [43], [44] that were observed in *Bmi1* mutant mice. Pietersen AM *et al.* reported that co-deletion of the *Ink4a/Arf* locus can rescue severe mammary-epithelium growth defects observed in Bmi1 deficient mice [45]. In the present study, we found that double deletion of *Bmi1* and *Ink4a/Arf* in mice rescued the oval cell expansion defects induced by the loss of *Bmi1*. Therefore, our data imply that Bmi1, similar to that described in other tissue types, regulates hepatic oval cell expansion in an *Ink4a/Arf*-dependent manner.

While our studies showed that Bmi1 is required for hepatic progenitor cell expansion, it remains unknown whether Bmi1 is required for adult hepatocyte proliferation. To investigat this question, we performed 2/3 partial hepatectomy on *Bmi1^−/−^* mice. However, none of the *Bmi1^−/−^* mice survived the surgery and all animals died within 24 hours. In contrast, all control *Bmi1^+/+^* and *Bmi1^+/−^* mice were still alive 48 to 72 hours post surgery (Fan L, unpublished observation). This is likely due to the severe hematopoietic defects characteristic of *Bmi1^−/−^* mice. Thus, we used an alternative approach by comparing the proliferative rates of hepatocytes from 2-week-old young mice, when hepatocytes are actively proliferating, in *Bmi1^−/−^* and *Bmi1^+/+^* mice. Brdu incorporation assay and Ki67 staining were used to assess hepatocyte proliferation. We found that there was no significant difference between *Bmi1^−/−^* and their littermate control mice (Figure S5). The data suggest that it is likely Bmi1 is not required for hepatocyte proliferation. However, more studies are required before the definitive conclusion can be reached on this issue. The most appropriate approach is to generate liver specific *Bmi1* knockout mice by crossing *AlbCre* mice [46] with *Bmi1^flox/flox^* mice [47] Partial hepatectomy should be performed on these conditional knockout mice, and whether Bmi1 is required for hepatocyte proliferation can be definitively determined

Liver cancer has two major types, namely HCC and CC, which are believed to be derived from hepatocytes and cholangiocytes, respectively. However, the concept of hepatic progenitor cells as targets of hepatocarcinogenesis has recently been elicited [48], [49], [50], even though it is still quite controversial. Increasing evidence indicated the link between hepatic oval cells and HCC/CC mixed liver tumors. For example, in a recent study by Samira *et al.*, a progressive expansion of oval cells was induced by liver specific deletion of *Nf2* (*neurofibromatosis type 2*) tumor suppressor gene. Importantly, all those mice eventually developed both HCCs and CCs, suggesting that *Nf2^−/−^* progenitors can be a cell of origin for these tumors [51]. In another study by Lee *et al.*, a similar HCC/CC mixed phenotype was developed in mice heterozygous for the tumor suppressor WW45 or in mice with liver-specific WW45 ablation. All the tumors were positive for the oval cell marker A6, supporting the progenitor cell origin of the mixed HCC/CC tumor cells [46].

In a recent study, we developed a novel mouse model in which HCC/CC combined liver tumors were developed by co-activating AKT and N-Ras oncogenes via hydrodynamic injection [15]. We now show that the AKT/Ras tumor cells overexpressed the oval cell marker A6. The expansion of cells positive for the A6 oval cell marker is not universally detected in mouse liver tumor models. For instance, we found that in other liver tumor mouse models, such as those induced by injecting c-Myc or c-Met/β-catenin, the A6 expression was restricted to the bile duct cells, and not observed in tumor cells (Fan L, unpublished observation). Thus, it is possible that AKT/Ras co-expression induces liver tumor development via expansion of hepatic progenitor cells or converting hepatocytes into progenitor-like cells during malignant transformation. By combining the AKT/Ras tumor model and *Bmi1* null mice, we investigated the role of Bmi1 during hepatocarcinogenesis induced by AKT/Ras overexpression. Our results show that ablation of *Bmi1* dramatically decreases the tumor progression in AKT/Ras mice. Indeed, AKT/Ras control mice required to be euthanized 5 to 7 weeks post hydrodynamic injection due to the liver tumor burden, while the *Bmi1* null mice overexpressing AKT/Ras developed only lipid-rich preneoplastic lesions, with no visible signs of malignancy. Of note, the absence of frankly malignant tumors was paralleled by the reduced expression of the A6 oval cell marker in *Bmi1* null mice. Thus, these data suggest that Bmi1 play an important role in AKT/Ras hepatocarcinogenesis, most likely via regulating hepatic progenitor cell proliferation. Furthermore, as we have shown that Bmi1 regulates hepatic oval cell expansion via regulating the *Ink4A/Arf* locus, it would be important to determine whether loss of *Ink4A/Arf* locus can rescue the tumor phenotype in *Bmi1* null mice. This experiment is currently in process and will be reported separately.

Our previous studies showed Bmi1 cooperates with activated Ras pathways to promote hepatic carcinogenesis *in vivo* [28]. In addition, a recent study by Chiba T *et al* demonstrated that overexpression of Bmi1 in *Ink4a/Arf* ^−/−^;Dlk(+) liver progenitor cells led to tumor formation in the Xenograft model [52]. Our current study adds to all these previous studies, providing further evidence that Bmi1 functions as an oncogene and is required for liver cancer development. Altogether, these studies suggest that targeting Bmi1 may be a novel therapeutic strategy for the treatment of liver cancer.

## Materials and Methods

### Constructs and reagents

All the constructs, including pT3-EF1α-RasV12, pT3-EF1α-myr-AKT and pCMV-SB used for mouse injection were previously described [34], [35], [36]. DDC was purchased from Deans Animal Feed Inc. (Redwood City, CA) and NAC from Sigma-Aldrich (St. Loise, MO).

### Mice

*Ink4a/Arf^−/−^* mice were purchased from the NCI Mouse Models of Human Cancers Consortium (MMHCC). *Bmi1^+/−^* mice were kindly provided to us by Dr. Carla Kim of Harvard University. *Bmi1^+/−^* mice were intercrossed to generate *Bmi1* mutant mice. *Ink4a/Arf^−/−^* mice and *Bmi1^+/−^* mice were mated and the offspring were backcrossed to generate double mutant mice (*Bmi1^−/−^; Ink4a/Arf^−/−^*). Briefly, *Bmi1^+/+^; Ink4a/Arf^−/−^* mice and *Bmi1^+/−^; Ink4a/Arf^+/+^* mice were initially mated to generate *Bmi1^+/−^; Ink4a/Arf^+/−^* mice. These mice were further mated with *Bmi1^+/+^; Ink4a/Arf^−/−^* mice to generate *Bmi1^+/−^; Ink4a/Arf^−/−^* mice. Finally, *Bmi1^+/−^; Ink4a/Arf^−/−^* mice were intercrossed to generate *Bmi1^−/−^; Ink4a/Arf^−/−^* and *Bmi1^+/+^* or *Bmi1^+/−^; Ink4a/Arf^−/−^* littermates. Genotyping was performed by polymerase chain reaction (PCR) on genomic DNA from tail clips (list of primers are available upon request). Sulfamethoxazole and trimethoprim combination (TMS) was added to drinking water at 1∶40 ratio to prevent potential infection in *Bmi1* mutant mice.

For hepatic oval cell expansion, 6-week-old mice were supplied, continuously for 3 weeks, with a diet containing 0.1% DDC. For the *in vivo* administration of NAC, water containing NAC at 1 mg ml^−1^ (0.1%) was supplied to animals starting from at 5weeks of age (one week before DDC treatment) and administration of NAC continued throughout the DDC treatment course. To examine the hepatocyte proliferation, Brdu (100 mg/kg body weight) was injected i.p. 2 hours before the mice were euthanized.

For the mouse liver tumor model, *Bmi1^+/−^* mice were backcrossed with wild-type FVB/N mice obtained from Charles River (Wilmington, MA) for five passages. The *Bmi1^+/−^* mice were inter-crossed to obtain *Bmi1^−/−^* mice as well as control littermates. The hydrodynamic injection procedures were performed as previously described [36]. In brief, pT3-EF1α-RasV12, pT3-EF1α-myr-AKT, and pCMV-SB were mixed at the ratio of 25∶25∶2 in saline and injected at 1/10 volume of mouse weight in 5 to 7 seconds.

All mice were housed, fed and treated in accordance with protocols approved by the committee for animal research at the University of California, San Francisco.

### Immunohistochemistry and Immunoflourescence

For immunohistochemistry staining, liver tissue was divided and fixed in 4% paraformaldehyde overnight at 4°C, then in 75% ethanol overnight at 4°C and processed to be embedded in paraffin blocks. Paraffin slides were dewaxed by xylene, followed by rehydrating through a series of washes with incrementally decreasing percentages of ethanol. Antigen retrieval was performed in 10 mM sodium citrate buffer (pH 6.0) by placement in a microwave on high for 10 min, followed by a 20-min cool down at room temperature. After a blocking step with the 5% goat-serum and Avidin-Biotin blocking kit (Vector Laboratories, Burlingame, CA). The slides were incubated with a primary antibody: anti-Ki67 (Labvision, Fremont, CA) at a 1∶150 dilution overnight at 4°C; anti-Bmi1 (Bethyl) at a 1∶100 dilution overnight at room temperature. Slides were then subjected to 3% hydrogen peroxide for 10 min to quench endogenous peroxidase activity and subsequently the biotin conjugated secondary antibody at a 1∶400 dilution for 30 min at room temperature. Detection was performed with the ABC-Elite peroxidase kit (Vector Laboratories) by using the DAB substrate kit (Dakocytomation).

For immunoflourescence staining, liver tissue was freshly isolated from euthanized animals and directly embedded in O.C.T. compound and frozen in cold 2-Methylbutane. Frozen sections were cut at 5 µm, blocked with 5% goat serum, labeled with primary antibodies (A6, CK19, OC2-2A6) at 1∶200 dilution overnight at 4°C and secondary antibody Alexa Fluor®594 or Alexa Fluor®488 goat anti-rat IgG (Invitrogen, Carlsbad, CA) at 1∶500 dilution for 30 min at room temperature. A6 antibody was kindly supplied by Dr. V. Factor (Laboratory of Experimental Carcinogenesis, NCI, NIH). OC2-A6 antibody was a generous gift from Dr. Markus Grompe (Oregon Health and Science University). CK19 antibody was purchased from Developmental Studies Hybridoma Bank (Iowa City, IA). For Brdu staining, two additional steps of 30-min incubation with 2N HCl at 37°C followed by a 10-min rinsing in 0.1 M borate acid buffer at room temperature were performed before incubation with primary antibody (anti-Brdu, 1∶100, Labvision). The immunofluorescence signal was visualized by a immunofluorescence microscope after the sections were mounted with VECTASHIELD® Mounting Medium with DAPI (Vector Laboratories, Inc). Antibody negative controls were performed by omitting the primary antibody from the protocol.

### Quantitative Real-time RT–PCR

Sybergreen based quantitative real-time RT-PCR was performed using SYBR Green master mix (Life Technologies, Grand Island, NY) according to the manufacturer's protocol. Primer sequences are the same as previous published [28].

### Malondialdehyde (MDA) Assay

Formation of MDA in the mouse liver tissues were analyzed using the Thiobarbituric Acid Reactive Substances (TBARS) kit (Cayman Chemical Company, Ann Arbor, MI).

### Data Analysis

The threshold of positive staining area related to positive staining nuclei by DAPI was measured by ImageJ software (http://rsb.info.nih.gov/ij/). Three randomly selected areas of each slide were analyzed at 40× magnification. Each experiment was repeated at least three times and data were expressed as means ± standard deviation (SD). Student's t test was used to evaluate statistical significance. Values of *P*<0.05 were considered to be statistically significant.

## Supporting Information

Figure S1 **Representative immunohistochemistry of Bmi1 (A,C,E) and CK19 (B,D) in wild-type mouse livers treated with DDC.** Note that both antibodies stain cells located in the liver periportal regions. As shown in (E), these cells tend to form pseudo-ductular structures (thin arrows) and are morphologically distinct from surrounding hepatocytes (thick arrows), exhibiting the prototypical features of oval cells (small and oval nuclei).
(TIF)

Figure S2 **Bmi1 is overexpressed in oval cells expanded liver. Relative expression of mBmi1 in Bmi1 WT livers without DDC treatment (n = 3) and with 3 weeks DDC treatment (n = 3).**
(TIF)

Figure S3 **ROS activation assessed by MDA content was not elevated in Bmi1 KO mice (n = 3) livers compared with Bmi1 WT mice (n = 3) livers after 3 weeks DDC treatment.**
(TIF)

Figure S4 **Ink4a/Arf encoded P16 and P19 are increased in oval cells expanded liver.** Relative expression (Log2) of mP16 (A) and mP19 (B) in Bmi1 WT livers (n = 3) and Bmi1 KO livers (n = 3) after 3 weeks DDC treatment.
(TIF)

Figure S5 **Loss of Bmi1 does not affect hepatocytes proliferation.** (A) Ki67 (upper) and Brdu (lower) staining of hepatocytes of 2-weeks-old Bmi1 WT and KO mouse liver. Representative images of three independent experiments are shown. (B) Quantification of Ki67 and Brdu staining. At least three random fields of each section were counted for quantification and a total of three batches of mice were analyzed.
(TIF)
