# Supplementary figures and images for: Bmi1 Is Required for Hepatic Progenitor Cell Expansion and Liver Tumor Development
Source: PLoS One. 2012 Sep 28;7(9):e46472. doi: 10.1371/journal.pone.0046472 (PMC3460872; doi:10.1371/journal.pone.0046472)

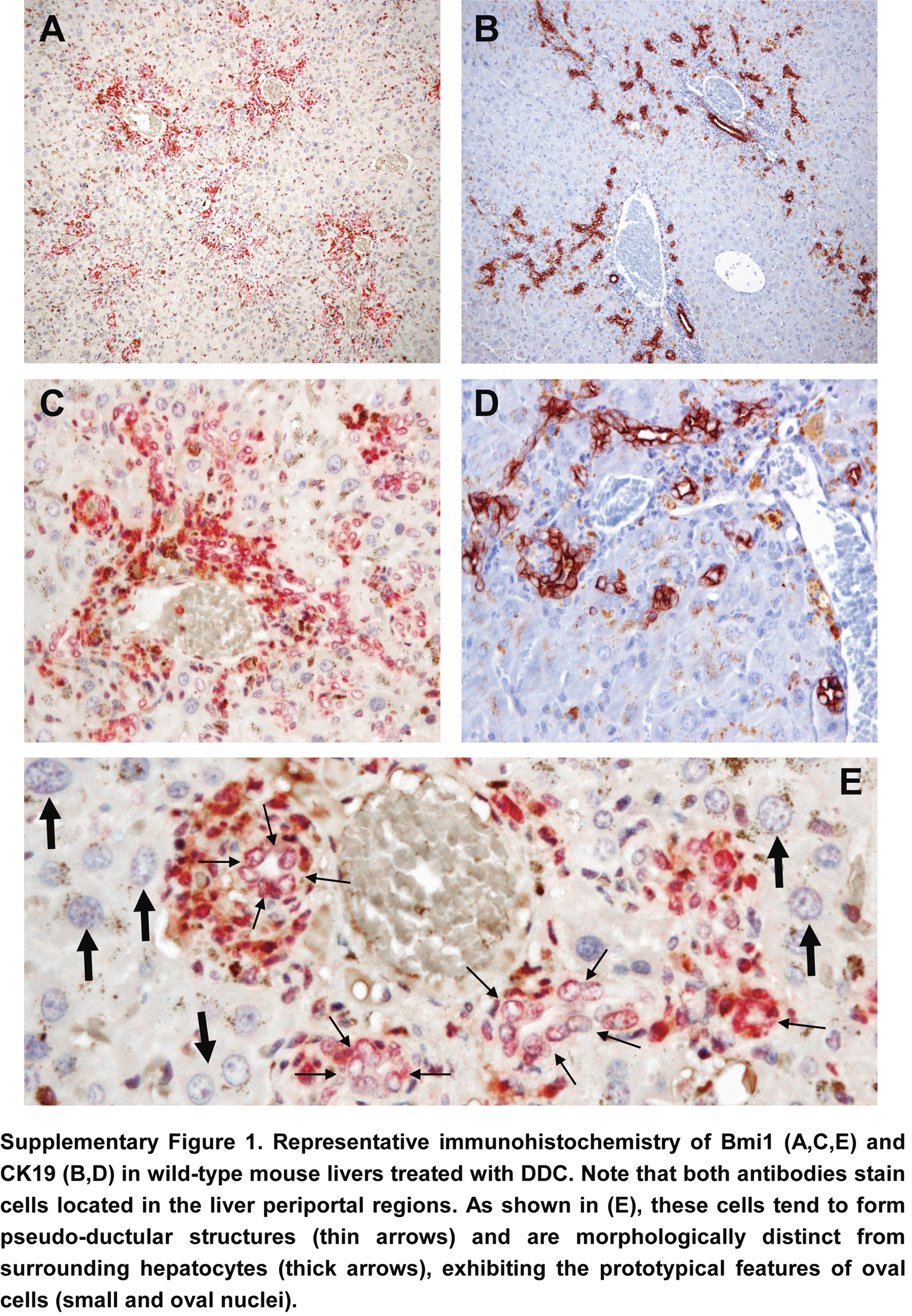

Supplement: Figure S1 — Representative immunohistochemistry of Bmi1 (A,C,E) and CK19 (B,D) in wild-type mouse livers treated with DDC. Note that both antibodies stain cells located in the liver periportal regions. As shown in (E), these cells tend to form pseudo-ductular structures (thin arrows) and are morphologically distinct from surrounding hepatocytes (thick arrows), exhibiting the prototypical features of oval cells (small and oval nuclei). (TIF) [file pone.0046472.s001.tif]

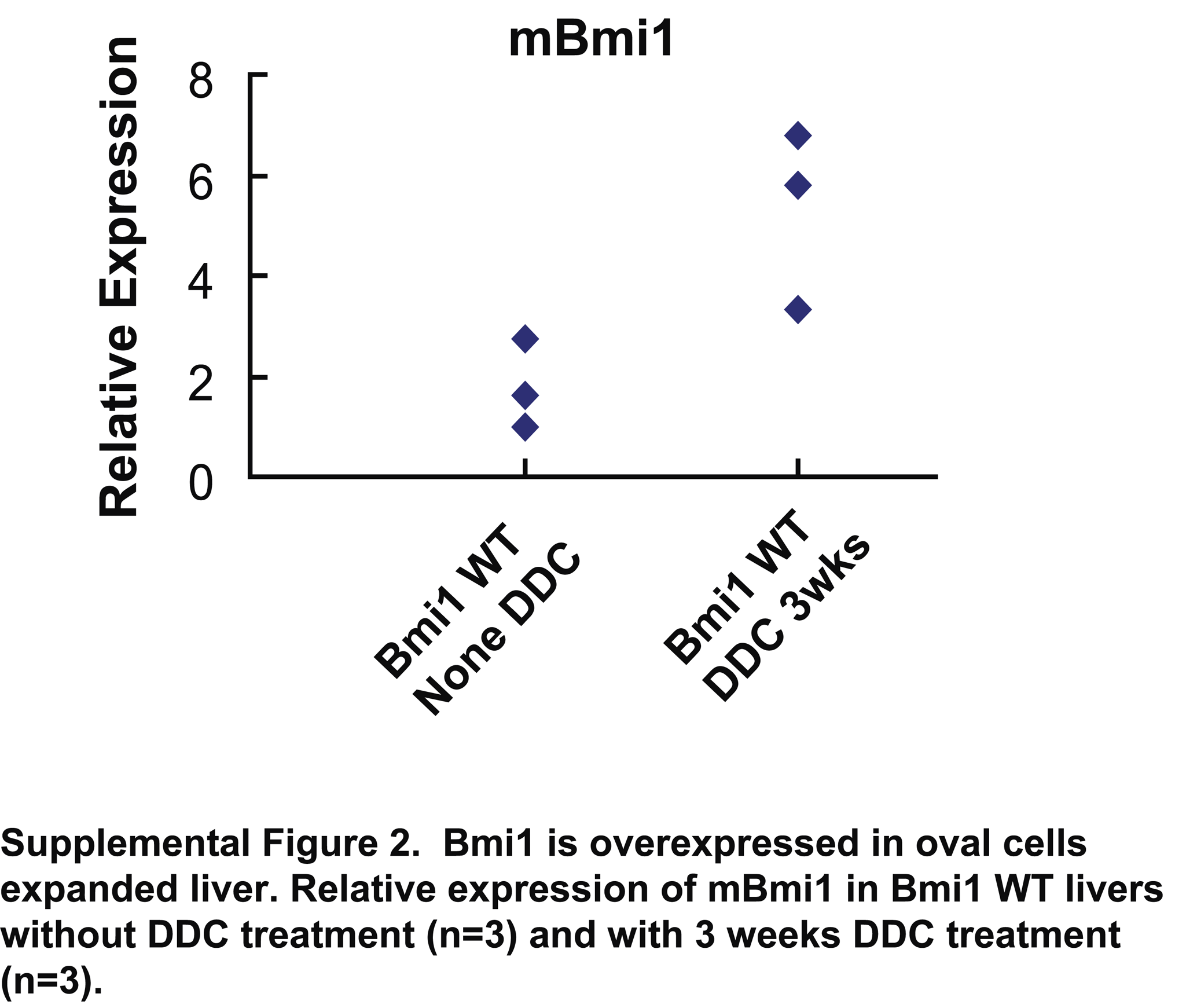

Supplement: Figure S2 — Bmi1 is overexpressed in oval cells expanded liver. Relative expression of mBmi1 in Bmi1 WT livers without DDC treatment (n = 3) and with 3 weeks DDC treatment (n = 3). (TIF) [file pone.0046472.s002.tif]

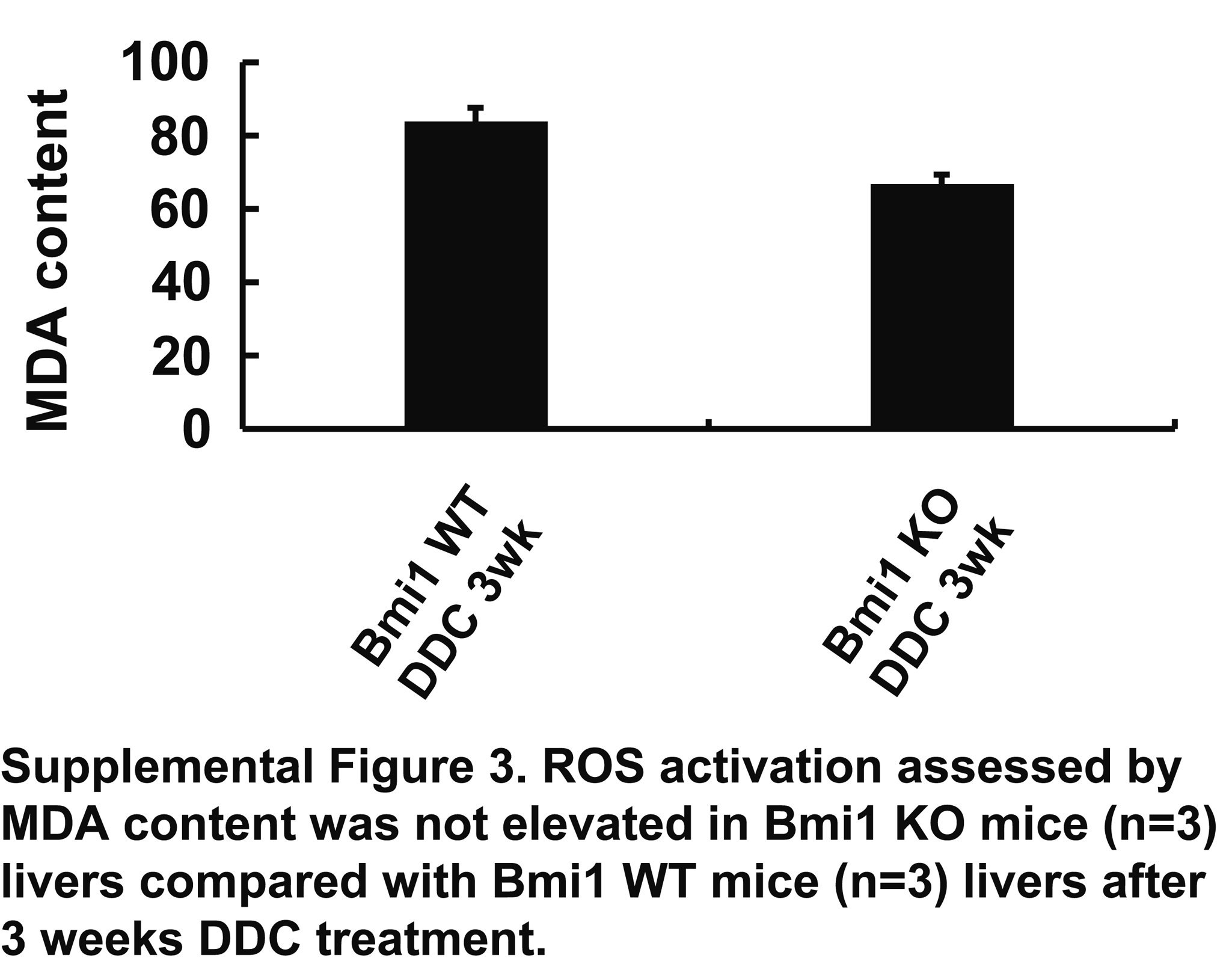

Supplement: Figure S3 — ROS activation assessed by MDA content was not elevated in Bmi1 KO mice (n = 3) livers compared with Bmi1 WT mice (n = 3) livers after 3 weeks DDC treatment. (TIF) [file pone.0046472.s003.tif]

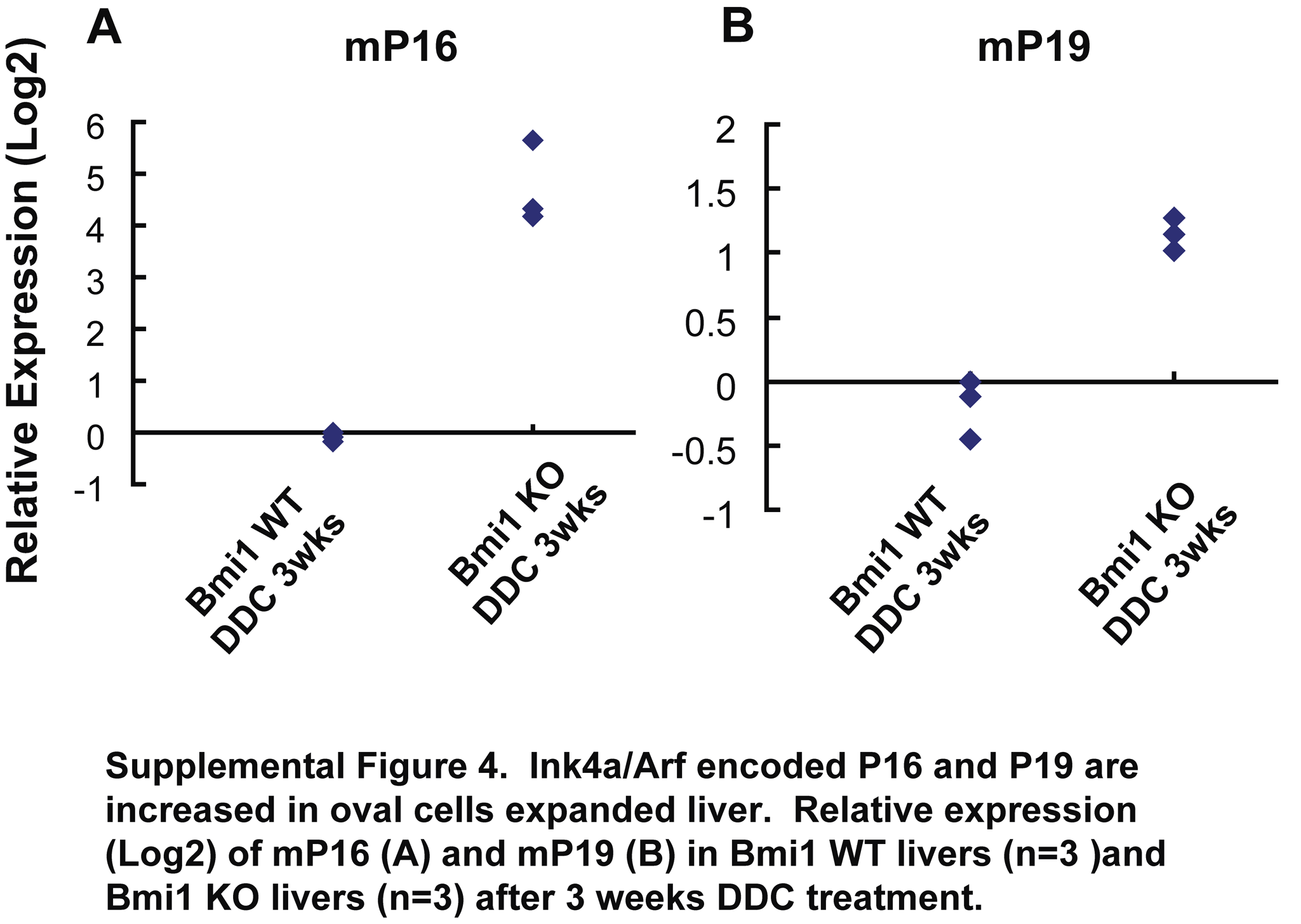

Supplement: Figure S4 — Ink4a/Arf encoded P16 and P19 are increased in oval cells expanded liver. Relative expression (Log2) of mP16 (A) and mP19 (B) in Bmi1 WT livers (n = 3) and Bmi1 KO livers (n = 3) after 3 weeks DDC treatment. (TIF) [file pone.0046472.s004.tif]

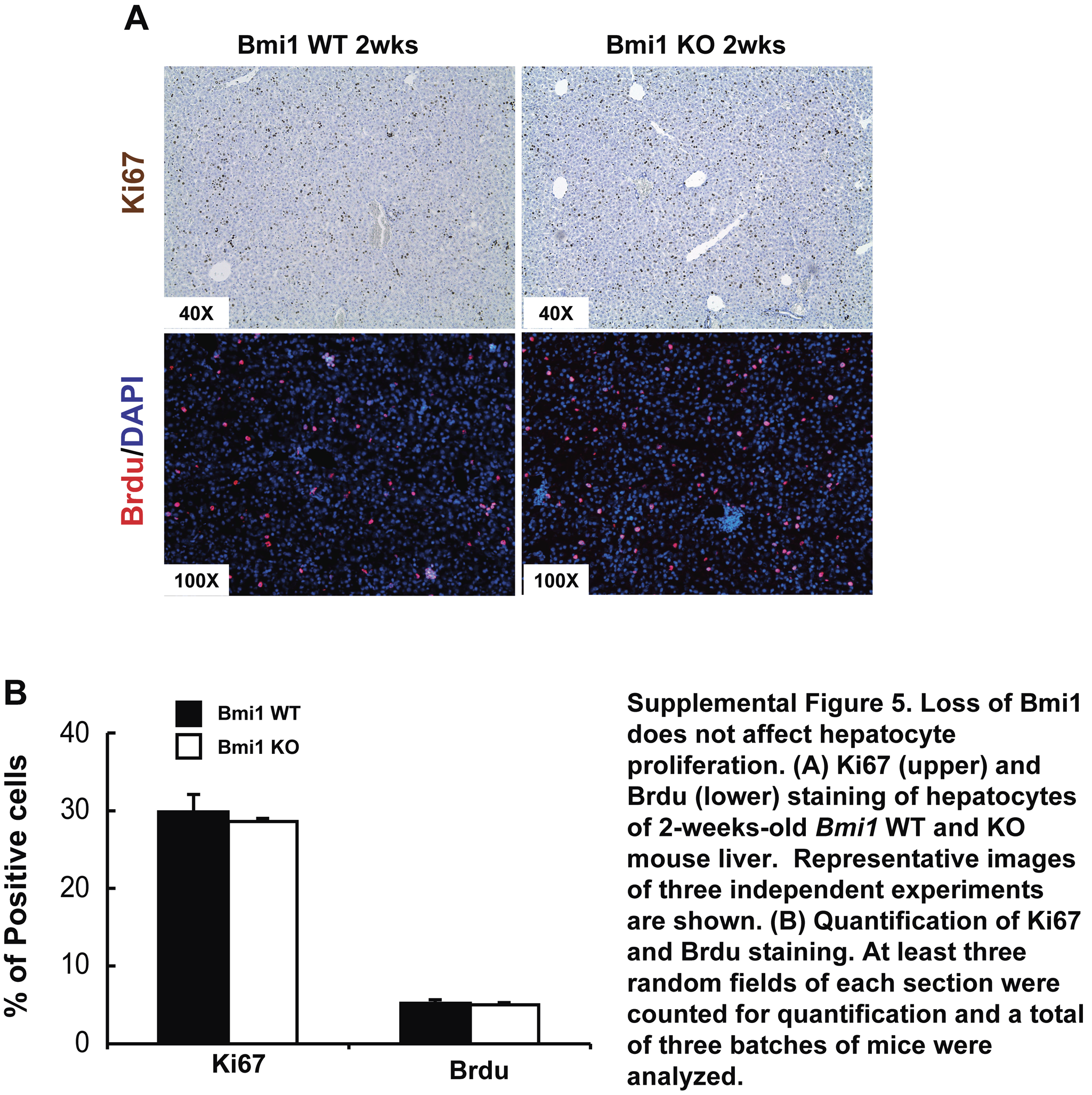

Supplement: Figure S5 — Loss of Bmi1 does not affect hepatocytes proliferation. (A) Ki67 (upper) and Brdu (lower) staining of hepatocytes of 2-weeks-old Bmi1 WT and KO mouse liver. Representative images of three independent experiments are shown. (B) Quantification of Ki67 and Brdu staining. At least three random fields of each section were counted for quantification and a total of three batches of mice were analyzed. (TIF) [file pone.0046472.s005.tif]
